# Photostable Iridium(III) Cyclometallated Complex is an Efficient Photosensitizer for Killing Multiple Cancer Cell Lines and 3D Models under Low Doses of Visible Light

**DOI:** 10.1021/acs.jmedchem.4c00869

**Published:** 2024-09-04

**Authors:** Callum Jones, Marta Martinez-Alonso, Hannah Gagg, Liam Kirby, Julia A. Weinstein, Helen E. Bryant

**Affiliations:** † School of Medicine and Population Health, University of Sheffield, Beech Hill Road, Sheffield S10 2RX, U.K.; ‡ Department of Chemistry, University of Sheffield, Sheffield S3 7HF, U.K.

## Abstract

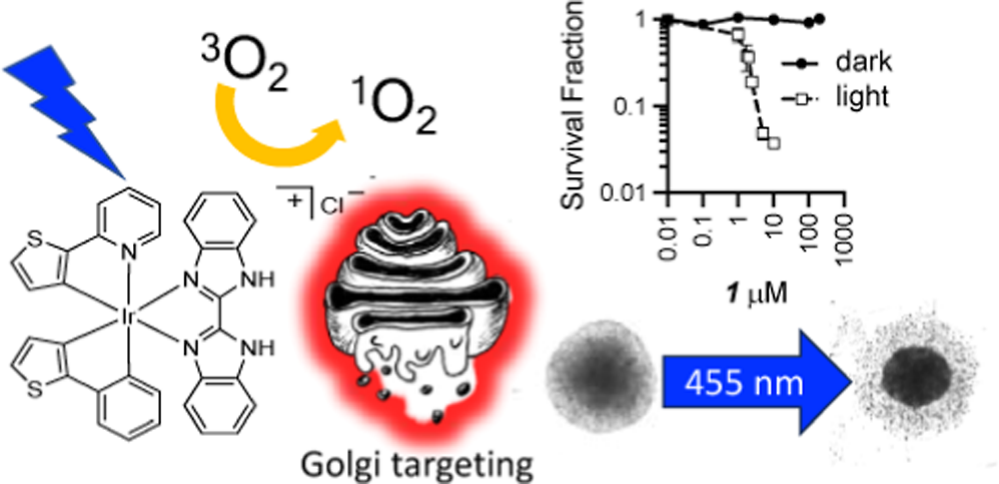

Photodynamic therapy delivers more targeted cell killing than classical chemotherapy. It uses light-absorbing compounds, photosensitizers (PSs), to generate lethal reactive oxygen species (ROS) at sites of localized irradiation. Transition metal complexes are attractive PSs due to their photostability, visible-light absorption, and high ROS yields. Here, we introduce a low-molecular weight, photostable iridium complex, [Ir(thpy)_2_(benz)]Cl, **1**, that localizes to the Golgi apparatus, mitochondria, and endoplasmic reticulum, absorbs visible light, phosphoresces strongly, generates ^1^O_2_ with 43% yield, and undergoes cellular elimination after 24 h. **1** shows low dark toxicity and under remarkably low doses (3 min, 20–30 mJ s^–1^ cm^–2^) of 405 or 455 nm light, it causes killing of bladder (EJ), malignant melanoma (A375), and oropharyngeal (OPSCC72) cancer cells, with high phototoxic indices > 100–378. **1** is also an efficient PS in 3D melanoma spheroids, with repeated short-time irradiation causing cumulative killing.

## 1 Introduction

Photodynamic therapy (PDT) works through the application of a prodrug, termed a photosensitizer (PS), which induces cell death upon light irradiation through the generation of reactive oxygen species (ROS).^1,2^ The PS should be non-toxic without irradiation, reducing off-target cell damage and achieving site-specific effects via tumor-localized irradiation. Absorption of light by a molecule of the PS populates its singlet excited state, ^1^PS*, which undergoes intersystem crossing to a triplet excited state, ^3^PS*. This state subsequently interacts with endogenous cellular oxygen in a bimolecular reaction, generating singlet oxygen (^1^O_2_) and/or other ROSs. ^1^O_2_ is a very strong oxidant that induces oxidative damage to biological entities such as proteins, lipids, and nucleic acids, leading to irreparable cellular stress within the subcellular organelle to which the PS and irradiation are targeted.^3,4^ Whilst a multitude of organic-based PS is known, there are still significant limitations due to long clearance times, poor solubility, and especially photobleaching (decomposition of PS under the light used).^5,6^ Transition metal complexes are potentially efficient PSs due to their strong visible-light absorptions, high yields of ^1^PS* → ^3^PS* due to the “heavy atom effect” of the metal center enhancing intersystem crossing, and long excited-state lifetimes which ensure high yields of ^1^O_2_.^7,8^ Transition metal complexes also offer unparalleled versatility of structure and tunability of their properties, by varying, in a modular fashion, the metal and the ligands and/or by modifying the periphery of the complex without altering the key photophysical properties. Such versatility can increase efficiency of the PS by shifting its absorption into the red(er) region of the spectrum which has better tissue transparency, by increasing their solubility, or by specific organelle targeting.^9,10^ Furthermore, the long-lived phosphorescence of transition metal complexes offers their dual use as bioimaging and therapeutic agents.^11^ Reported transition metal complexes as PS include among others, those of Ru, Pt, and Os.^12,13^ The most striking example, a Ru(II) TLD1433, has entered phase-II clinical trials for the treatment of non muscle invasive bladder cancer.^14,15^

Exploring the potential of transition metal-based PS as imaging and PDT agents, we have previously shown that a cyclometallated Ir(III) complex, [Ir(ppy)_2_(benz)]^+^, where ppy = 2-phenyl-pyridine, benz = 2,2′-bisbenzimidazole, is an efficient cell-killing agent activated by 405 nm light.^8^ Here, we present a novel Ir(III) complex of the family [Ir(N̂C)_2_(NN)]^+^, [Ir(thpy)_2_(benz)]Cl, **1**, which is active under visible light. In **1**, the 2-phenyl group is replaced by a more electron-donating 2-thienyl. In such complexes, the lowest absorption bands have significant N̂C intraligand charge-transfer character, whereby electron density is shifted to the pyridine ring (acceptor) from the cyclometallating ring (donor). By introducing a more electron-rich cyclometallating thienyl fragment, the relevant absorption bands shift to lower energies, into the visible region. We demonstrate that this subtle change in design leads to an efficient PS which has a high phototoxic index (PI) at low light doses and short illumination times, not only under 405 nm but also the more tissue-penetrating visible light (455 nm). **1** is not toxic without light, accumulates rapidly in live cells, localizes to the mitochondria, endoplasmic reticulum (ER), and Golgi apparatus, and is eliminated from cells after24 h. The strong phosphorescence of **1** opens its dual use as an imaging and therapeutic agent. We also show that **1** is an efficient PS in 3D spheroids, operating at low doses and short illumination times. In 3D spheroids, it also demonstrates fast elimination and shows cumulative action during repeated illuminations.

## 2 Results and Discussion

### 2.1 Crystal Structure and Photophysical Properties of **1**

**Figure 1:**
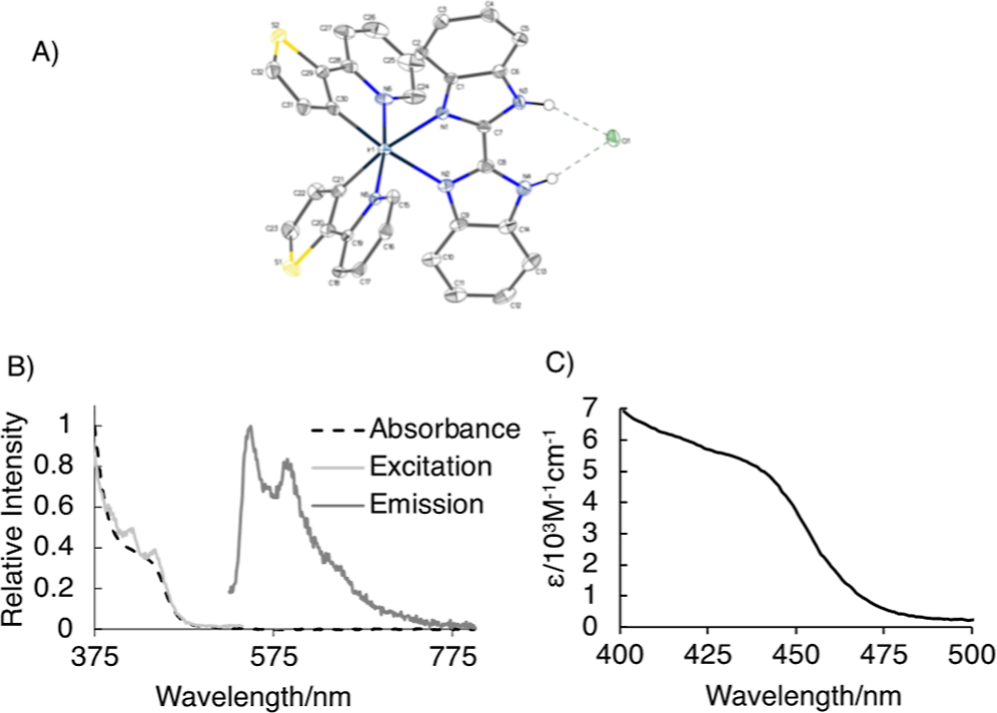
(A) Crystal structure of **1** determined by X-ray diffraction studies, CCDC2263098. (B) Absorption, emission (λ_exc_ 455 nm), and excitation (λ_em_ 550 nm) spectra of **1** in aerated DCM at rt. (C) Visible-light absorbing molar absorption coefficients, ε, of **1** in DMSO. The absorption and emission spectra of the complex did not change over the course of several days in DMSO, DCM, nor in the 1% DMSO/PBS.

Single crystals of **1** suitable for X-ray diffraction were grown by slow diffusion of hexane into a saturated solution of **1** in DCM, the resulting structure is shown in Figure 1A (Figure S4 and Tables S1–S8). The Ir–N bond lengths (2.05–2.18 Å) are characteristic for Ir complexes of this type and have a skewed octahedral geometry with the bite angle N–Ir–N of 76.2° at the coordinated benz ligand.^16,17^

The absorption spectrum of **1** (Figure 1B) contains bands in the UV region assigned to the intraligand transitions, while the broad, less intense absorption band at around 450 nm is due to ^1^MLCT Ir → benz, mixed with intraligand thienyl → pyridine charge-transfer transitions, which is typical in such complexes. Introduction of a stronger thienyl donor in thpy vs phenyl in the ppy ligand achieved the aim of red-shifting the absorption in the visible region up to 475 nm, with appreciable molar extinction coefficients, ε (7100 M^–1^ cm^–1^ at 400 nm, 2800 M^–1^ cm^–1^ at 455 nm). Upon excitation into the lowest absorption band of **1** in DCM, a strong yellow-orange emission was observed, centered at 550 nm; the broad emission band with pronounced vibronic progression indicates a mixed ILCT/MLCT nature of the emissive state. The emission lifetime of **1** in aerated DCM of 330 ns confirms the triplet nature of the emissive state (Figure S5). The emission intensity and lifetime of **1** are increased threefold upon removal of oxygen, again confirming the triplet nature of the lowest excited state and opening the prospect of generating ^1^O_2_ (Figures 1 and S5). The quenching rate constant of ^3^PS* by oxygen, *k*_q_, was estimated as 1.6 × 10^9^ M^–1^ s^–1^ (by using the Stern–Volmer equation τ_0_/τ = 1 + *k*_q_τ_0_[O_2_], where [O_2_] in DCM = 1.23 mM, τ_0_ = 990 ns, and τ = 330 ns^18,19^). **1** efficiently sensitizes ^1^O_2_ in solution with 43% yield, as determined by direct detection of ^1^O_2_ emission at 1270 nm relative to the standard, perinaphthenone (Figure S5).

### 2.2 **1** is Rapidly Taken up by a Range of Cancer Cell Lines

**Figure 2:**
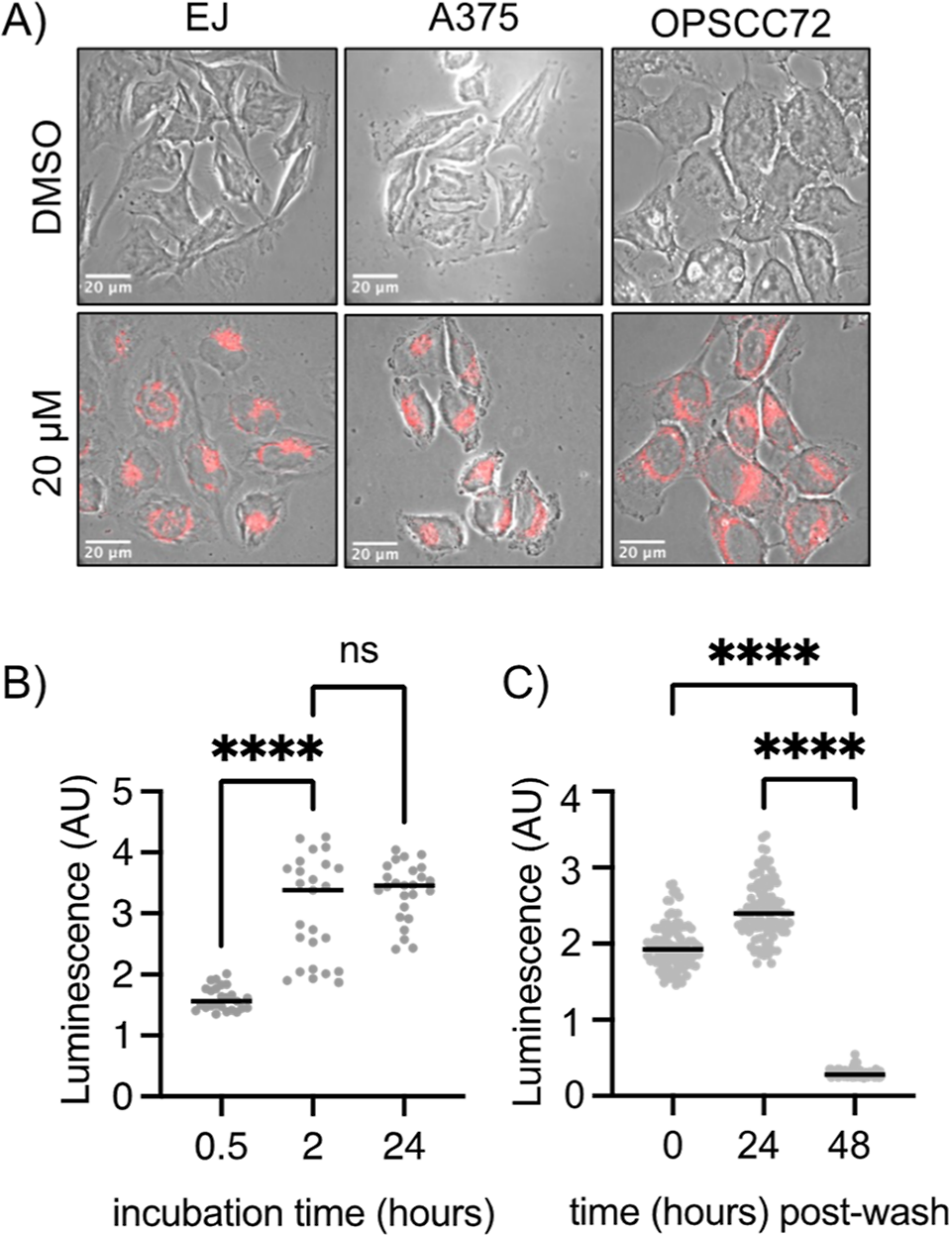
(A) Representative images of PDT-relevant cancer cell lines incubated with **1** (20 μM 0.4% DMSO/media, 2 h), scale bar = 20 μm. (B) Integrated luminescence intensity of **1** in EJ cells with increasing incubation times (*N* = 1, *n* = 25). (C) Integrated luminescence intensity of **1** in EJ cells after incubation (20 μM, 0.4% DMSO/media, 2 h) followed by washing and reincubation in untreated media for increasing times (*N* = 1, *n* = 100).

To evaluate the intracellular uptake, three cancer cell lines (bladder carcinoma, EJ; malignant melanoma, A375; and oropharyngeal cancer, OPSCC72) were exposed to a solution of **1** for various periods of time, washed, and imaged. The images (Figure 2A) show cytoplasmic staining, which is similar in all cell lines. The phosphorescence intensity in cells increased with the incubation time, maximizing at 2 h (Figures 2B and S6). The phosphorescence signal was retained for up to 24 h postincubation, after which it was no longer detectable (Figures 2C and S7). Thus, **1** is taken up rapidly and elicits good retention and elimination rates. Such properties are advantageous for PDT, allowing for PS activation in tissues while avoiding the unwanted accumulation of PS that can lead to side effects.^5,20^

### 2.3 **1** Localizes in Several Organelles and Disrupts the Golgi Apparatus when Illuminated

**Figure 3:**
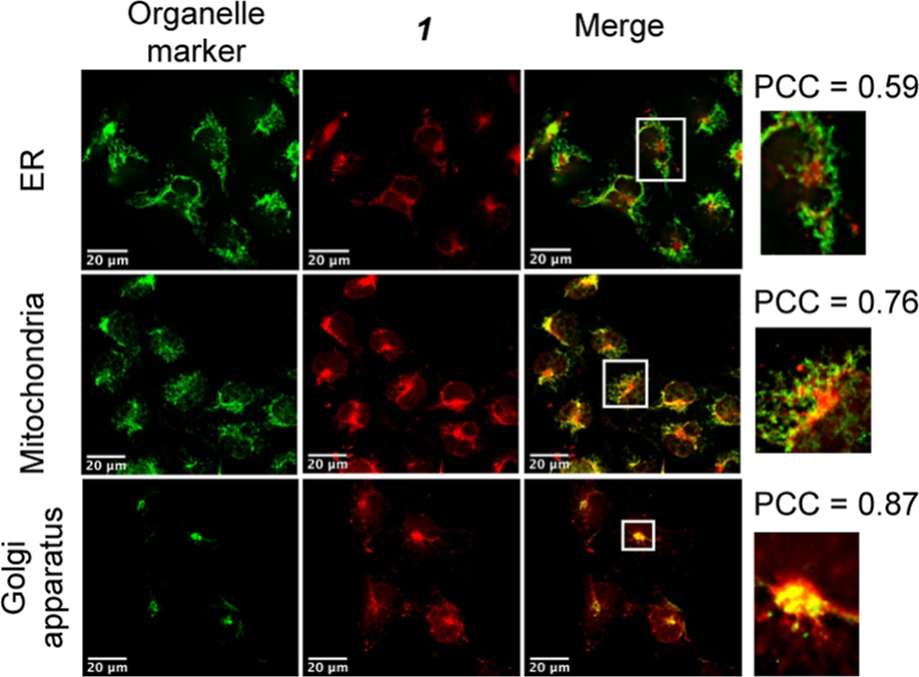
EJ cells following 2 h incubation with 30 μM **1** (red) (0.6% DMSO/media), colocalized with organelle-specific proteins (green). The luminescence overlap quantified as an average over 25 cells and colocalization expressed as a PCC (Pearson’s correlation coefficient). Zoomed sections are shown as insets. Scale bar = 20 μm.

Immunostaining in conjunction with visualization of the phosphorescence of **1** demonstrated that **1** targets the ER, mitochondria, and Golgi apparatus, suggesting that **1** may have use as an organelle imaging agent on fixed cells. The strongest colocalization signal was seen with components of the Golgi apparatus (Figure 3—Pearson’s correlation coefficient = 0.85, S8). Compared to other organelles, few Golgi-targeting small molecules are available.^21^ Illumination of **1**-incubated cells causes a change in the pattern of Golgi marker staining (Figure S9), whereby **1** + light, in comparison to **1** alone or light alone, caused widespread fragmentation immediately after the irradiation. This pattern is commonly reported as a marker of Golgi disruption, suggesting that **1** can induce photodamage of Golgi.^26^ Lesser colocalization of **1** with markers of the ER and the mitochondria were also detected (Figure 3). Given that photodamage at each of the three organelles triggers regulated cell death pathways such as autophagy and/or apoptosis,^22,23^ we propose that subcellular localization of **1** at these organelles is eliciting the potent phototoxic effects.

### 2.4 **1** Is an Efficient PS under 405 and 455 nm Light

**Figure 4:**
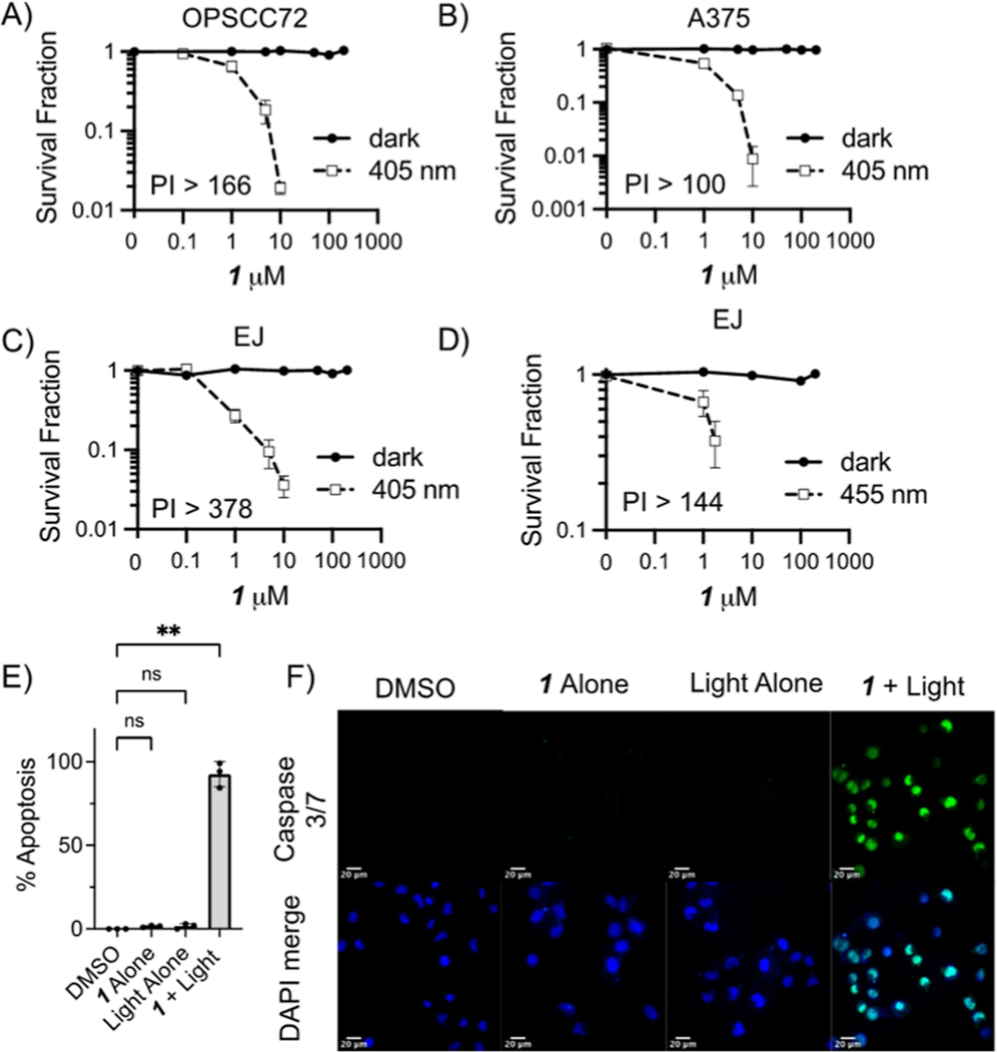
Clonogenic survival assays of PDT-relevant cancer cell lines after 2 h incubation in various concentrations of **1** followed by (A–C) 405 nm (20 mJ s^–1^ cm^–2^, 3 min) or (D) 455 nm (30 mJ s^–1^ cm^–2^, 3 min) irradiation. (E) Quantification and (F) representative images of caspase 3/7 activation (green) in EJ cells (blue) 24 h after treatment (2 h preincubation **1**, 10 μM 0.2% DMSO/media) followed by 405 nm (20 mJ s^–1^ cm^–2^, 3 min), scale bar = 20 μm. In each case, the mean and SD of 3 independent repeats are shown. ** indicates *p* < 0.01 (one-way ANOVA).

A range of cancer cell lines were incubated with **1** and their long-term clonogenic survival was assessed without irradiation (dark toxicity) and under 405 or 455 nm irradiation (light toxicity) (Figure 4A–D). In the absence of light, **1** did not induce cell killing up to 200 μM (the maximum concentration used was limited by the solubility of **1** and cellular DMSO toxicity). In contrast, exposure to **1** combined with mild irradiation with low power 405 nm light (20 mJ s^–1^ cm^–2^, 3 min) led to a dose-dependent reduction in cell survival in amelanotic melanoma,^24^ bladder carcinoma, and oropharyngeal cancer cell lines. PIs [LD_50_(dark)/LD_50_(light)] in the range of >100–378 demonstrate the potential of **1** as a powerful PS in a range of cancer cell lines [again, note that PI is the lower limit, as the PI estimate is based on solubility-limited LD_50_(dark)]. No significant cell killing was seen with light alone (Figure S10). One goal of PS development is to shift their absorption toward the red region of the spectrum, allowing for deeper tissue penetration of light. Clonogenic survival assays were repeated in bladder cancer cells at 455 nm (Figure 4D). Given a reduction in absorptivity at this wavelength (∼1/2 of 405 nm), the power used at 455 nm was increased slightly to 30 mJ s^–1^ cm^–2^ to enable comparison. This power did not result in any cell killing by light alone (Figure S10). Cell killing was observed in cells incubated with **1** under 455 nm irradiation with the value of PI > 144 demonstrating that, at this more tissue-penetrating wavelength, **1** still acts as an efficient PS.

The mechanism of light-activated cell killing induced by **1** + light was confirmed as apoptosis using a caspase 3/7 activation assay (Figure 4E,F), with little or no apoptosis in the cells treated with **1** alone or light alone. This mechanism is consistent with the observed disruption of the Golgi apparatus, mitochondria, and/or ER.^25,26^ An apoptotic death route is beneficial for wound healing and efficient immune clearance. While apoptosis was confirmed and from cell morphology appears to be the major route of cell death, one cannot exclude additional mechanisms of killing contributing to the overall efficacy.

### 2.5 PS Properties of **1** Translated to 3D Cancer Spheroid Models

**Figure 5:**
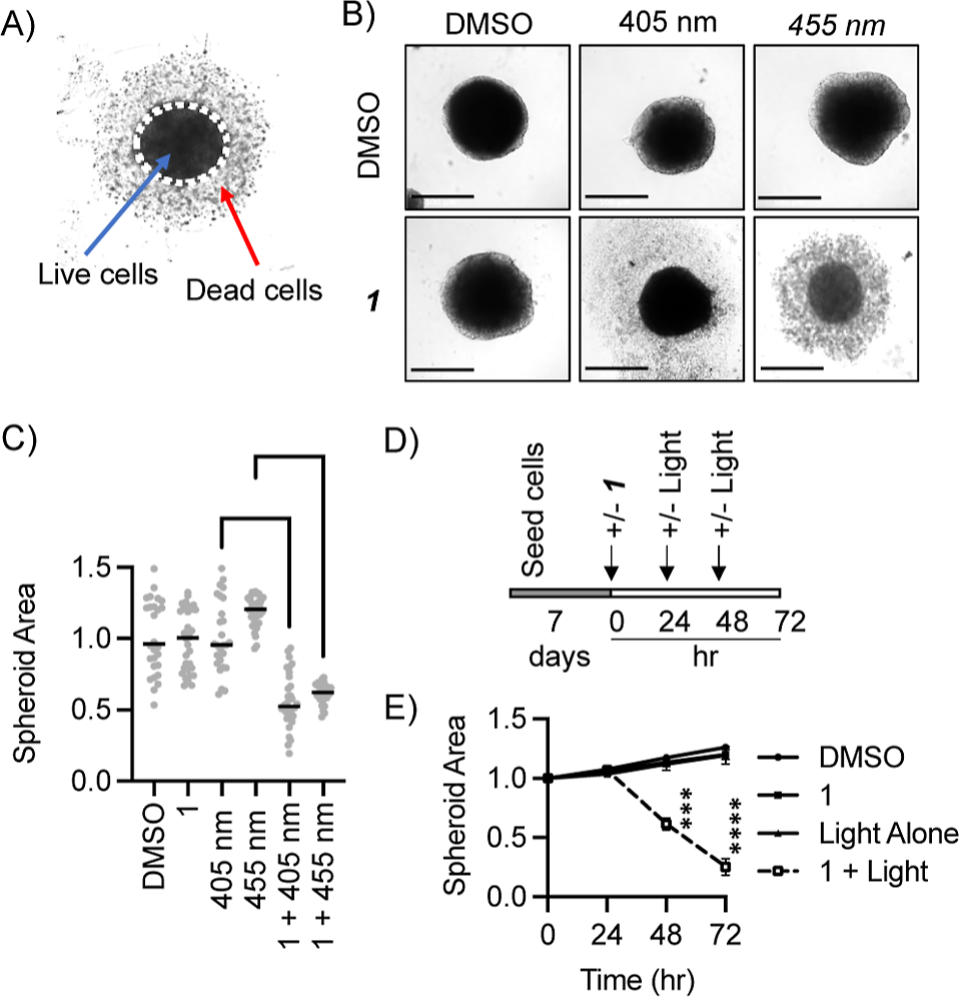
PS activity of **1** in a 3D model of metastatic melanoma. The spheroid area was calculated after incubation in **1** (10 μM, 0.2% DMSO/media) followed by 405 nm (20 mJ s^–1^ cm^–2^, 3 min) or 455 nm (30 mJ s^–1^ cm^–2^, 3 min) irradiation. (A) Representative image of C8161 spheroids treated by **1** + light shows live core (measured spheroid area) and surrounding dead cells. (B) Representative images of the spheroid area 48 h post single irradiation with 405 or 455 nm light in the presence or absence of **1**, scale bar = 500 μm, and (C) Quantification of pooled data from 3 independent repeats (12 replicates/repeat). Area is relative to pretreatment. Median spheroid size is indicated. **** = *p* < 0.0001 (Kruskal–Wallis test). (D) Schematic for double-light treatment assay, where the spheroid size was measured at 0, 24, 48, and 72 h. (E) Spheroid size relative to time 0 following single- or double-light treatment in the presence or absence of **1**. Mean and SD of 3 independent repeats (12 replicates/repeat) are shown. *** = *p* < 0.001 and **** = *p* < 0.0001 (2-tailed Student’s *t*-test).

2D cell monolayers lack spatio-heterogeneity, architecture, and communal growth observed in vivo.^27^ 3D-spheroid cell models offer a way to assess potential inhibitory barriers to PDT efficacy such as hypoxia, reduced drug/light penetration, or cell–cell interactions due to their heterogeneous structure.^28^ Amelanotic melanoma cells C8161^29−31^ were cultured as 3D spheroids, incubated with **1** and subjected to 405 or 455 nm light (Figure 5A–C). Spheroid size was reduced when **1** was activated by either wavelength of light, while spheroids subjected to light alone or **1** alone grew at a rate similar to that of untreated spheroids. Since, in a clinical setting, it is likely that sequential rounds of light therapy would be used, a second incubation and irradiation was performed. When spheroids were subjected to two rounds of light exposure, spheroid size was further reduced (Figures 5D,E and S11) compared to a single treatment. Each **1** + light treatment had an approximately equal effect on the spheroid size, reducing it by around 40%. Thus, **1** also shows high PS activity in 3D melanoma cell spheroid models, demonstrating further the potential of **1** for PDT. However, it should be noted that testing *in vivo* is required prior to any clinical translation.

## 3 Conclusions

A low-weight, visible-light-absorbing Ir(III) complex has been designed and shown as a potential PDT agent in cancer cell lines and spheroid models. A small change in design through the inclusion of the S-atom in the cyclometallating ligand, **1**, achieved an essential red shift in absorbance relative to the analogue without the S-atom while maintaining high levels of singlet oxygen production (43%). **1** was rapidly uptaken by a range of cancer cell lines, localizing to the Golgi apparatus, mitochondria, and ER, and clears from cells after 24 h. The photostability, lack of dark toxicity, and bright phosphorescence of **1** make it a novel fixed cell imaging agent capable of targeting multiple organelles. **1** induced high levels of photoactivated apoptotic cell death under low power (20–30 mJ s^–1^ cm^–2^) and short (3 min) irradiation at 405 or 455 nm. This light-induced cell killing was observed at ≤10 μM in a selection of cancer cell lines and in 3D spheroid models; with repeated short-term illumination having a cumulative effect. In contrast, **1** remained nontoxic in the dark up to a solubility-limited lower limit of 200 μM. The high PI (> 100–378), determined in all cases, demonstrates that **1** is a powerful PS that can operate in cells and 3D structures, under visible light, at low power densities and short irradiation times. The large therapeutic window in combination with the above characteristics makes this compound a promising PS agent.

## 4 Experimental Section

### 4.1 Synthesis of **1**

**Scheme 1:**
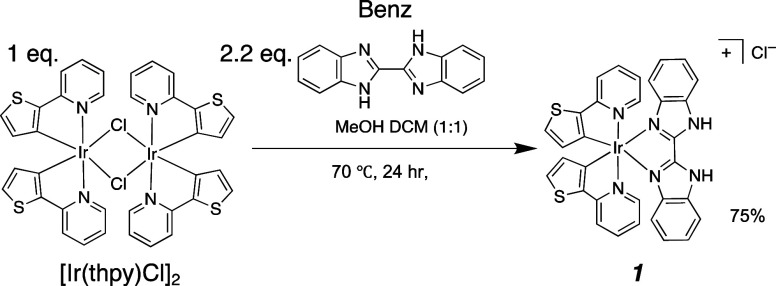
Synthesis of **1**

The new PS, **1**, where thpy = 2-thienyl pyridine, benz = 2,2′-bis-benzimidazole (Scheme 1), was synthesized from the chloride-bridged dimer [Ir(thpy)Cl]_2_ and benz following a procedure for analogous complexes.^8,32,33^ [Ir(thpy)Cl]_2_ (80 mg, 0.073 mmol, 1 molar equiv) and benz (37.2 mg, 0.16 mmol, 2.2 molar equiv) were placed under argon. Degassed methanol/DCM (1:1 v/v, 20 mL) was then added and the solid was dissolved to form an orange solution. The solution was then heated to 70 °C for 24 h under stirring and argon. The solution was cooled to room temperature and the solvent was removed under reduced pressure. The solid was redissolved in minimum DCM (∼5 mL) and then hexane (∼10–20 mL) was added to induce the precipitation of an orange solid. The solution was left overnight and the solid was collected by vacuum filtration (86.2 mg, 75%). The purity of the crystalline product >95% was confirmed by ^1^H and ^13^C NMR and mass spectrometry (Figures S1–S3).

### 4.2 Spectroscopy

UV/vis absorption spectra were all obtained on a Varian Cary 5000 UV–vis–NIR spectrophotometer with the pure solvent baselined to zero. The analyte in solution was measured in a quartz cuvette with a 1 cm path length. Emission and excitation spectra were all obtained on a FluoroMax4 spectrophotometer (HORIBA Jobin Yvon) with all solutions measured in quartz cuvettes with 1 cm path lengths. Time-decay lifetime measurements were all performed on a mini-t fluorescence lifetime spectrometer (Edinburgh Instruments) with a pulsed time modifiable laser diode source. Various filters were selected to measure the desired emission from the excited state.

### 4.3 Singlet Oxygen Determination

**1** and perinaphthenone were first dissolved in acetonitrile and diluted to give an absorbance value of 0.1 (±0.01) at 355 nm (the third harmonic of the Nd:YAG laser source). Singlet oxygen yield was calculated through excitation of an air-saturated solution of **1** in a 1 cm quartz cuvette through detection of the singlet to triplet oxygen relaxation emission (em = 1275 nm). The laser source used was a Q-SW Nd:YAG third harmonic 355 nm, 8 ns pulses (Ls-1231 M LOTISII 2006 model), and the emission was detected on an InGaAs photodiode with an Æ3 mm active area (J22D-M204-R03M-60-1.7, Judson Technologies). The photodiode used for excitation was coupled with a low-noise current amplifier (DLPCA-200, FEMTO Messtechnik GmbH), with the amplified signal recorded by a digital oscilloscope (TDS 3032B Tektronix). This setup effectively detects the decay signal of ^1^O_2_ into ^3^O_2_ with a high-contrast bandpass optical filter (1277 nm center wavelength, 28 nm fwhm, custom made by Izovac, Belarus) which is fitted onto the front of the detector. A singlet oxygen generation value was calculated by irradiating the sample containing either **1** or perinaphthenone (the reference). The decay signal of singlet oxygen was recorded and repeated 4 times, per power, and per solution and an average singlet oxygen decay signal was generated. The amplitude was then corrected by considering the exact optical density values at 355 nm (0.1 ± 0.01) using the following equation
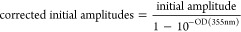
1

Equation 1: Calculation of the corrected initial amplitude to calculate singlet oxygen generation.

Singlet oxygen generation was calculated using the corrected initial amplitudes of both reference and **1** for each power value before an average is taken over the range of tested powers. The solvent was then taken into account due to potential quenching effects by reducing the amount of singlet oxygen present by a fixed amount depending on the exact system^34^
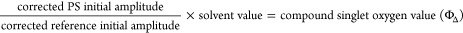
2

Equation 2: Determination of the singlet oxygen yield.

### 4.4 Cell Culture

Cells were routinely cultured and grown in suitable media (DMEM, 10% FCS) and incubated at 37 °C and 5% CO_2_. Cells were grown in a T-75 flask until reaching a suitable confluency, 80–100%. The media was drained from each flask followed by a PBS wash (10 mL) and detachment with trypsin–EDTA (1 mL). The cells were then resuspended in fresh media and counted on a hemocytometer with an average taken of the four quadrants. The stock solution was then diluted by a calculated amount and cells plated at a desired number in a suitable dish and incubated.

### 4.5 Microscopy

For widefield imaging, excitation was performed using a SpectraX LED source using the 470 nm or 395 nm wavelengths. The emission was captured by monitoring the relevant filter channels to specifically capture light from the target fluorophore. Images were collected as a series of z stacks using a 100× Ph oil (NA 1.4) lens. All images were processed by using FIJI imaging software (a version of ImageJ). For widefield z-stacked images, deconvolution was performed using the Richardson–Lucy algorithm to reduce out of focus light.

### 4.6 Cellular Uptake Assays

Cells were plated onto sterilized coverslips in 6 well plates (100,000–200,000 cells/well) and incubated overnight. The next day, the media was discarded and the cells were washed (3 × PBS, 1 mL/well) before addition of **1** (30 μM) and incubated for 30 min, 2 h, and 24 h. After this, each well was washed (3 × PBS, 1 mL/well), fixed (4% PFA in PBS, 10 min, rt), washed (3 × PBS, 1 mL/well), and finally mounted onto glass slides (ProLong Gold Antifade, 1 drop).

### 4.7 Cellular Elimination

Cells were plated onto sterilized glass coverslips in 6 well plates (50,000 cells/well) and incubated overnight at 37 °C. **1** was then added (30 μM, 2 h) to each well and incubated at 37 °C. After 2 h, the media was removed and each coverslip was washed (PBS, 1 mL/well) before being replenished with fresh media. After the time points, 0, 24, and 48 h, the coverslips had their media removed, were washed (3 × PBS, 1 mL/well), and then fixed (4% PFA in PBS, rt, 10 min). Each coverslip was then washed (3 × PBS, 1 mL/well) and then mounted onto glass microscope slides (ProLong Gold Antifade Mountant, 1 drop) before being allowed to dry and then stored in the fridge until imaged.

### 4.8 Uptake/Elimination Analysis

Each slide was imaged on a wide-field dual-cam Nikon live-cell microscope. The excitation and emission of the PS were imaged over 100 cells/condition (elimination) or 25 cells/condition (uptake). In ImageJ, the background-deducted cell-integrated fluorescence density was divided by the cell area to give an area accounted for by mean intensity. These values were measured under each condition and then plotted against each other to monitor PS uptake/elimination.

### 4.9 Colocalization Assays

Specific organelle staining was conducted using immunofluorescence performed by selecting a specific organelle-associated protein. The relevant primary antibody to these proteins was applied to form an antibody–protein conjugate which was visualized by the application of another relevant fluorophore-bound secondary antibody. Primary antibodies: mitochondria (anti-cytochrome c oxidase, 1:200 in PBS), ER (anti-calnexin, 1:100 in PBS), and the Golgi apparatus (anti-58K-9, 1:200 in PBS). Secondary antibodies; anti-mouse 488 1:500 in PBS and anti-rabbit 1:500 in PBS. Cells were plated onto glass coverslips at a density between 100,000 and 200,000/well in a 6-well dish and left overnight to adhere. **1** (30 μM, 2 h) was added to the relevant conditions and incubated at 37 °C. The media was then removed, and the coverslips were washed (3 × PBS, 1 mL/well) and then fixed (4% PFA in PBS, 10 min, rt, gentle agitation). Cells were then permeabilized (Triton X-100, 0.2% in PBS, rt, 10 min) and then washed (3 × PBS, 1 mL/well). Cells were then blocked (BSA 2% in PBS, rt, 1 h) and washed (3 × PBS, 1 mL). Coverslips were then inverted onto 100 μL of a relevant primary antibody and incubated in a humidified chamber at 4 °C overnight. The next day, coverslips were washed (3 × PBS, 1 mL/well) and inverted onto 100 μL of the relevant secondary antibody (1 h, rt). The coverslips were finally washed (3 × PBS, 1 mL/well) and then mounted with Prolong Gold Antifade onto glass microscope slides before being dried and stored at 4 °C until ready to image.

### 4.10 Colocalization Analysis

Microscopy was used to analyze the extent of emission overlap between **1**’s phosphorescence channel and the fluorescently labeled immuno-stained organelle channel. **1**-only and immuno-stained-organelle-only conditions were first imaged to ascertain experimental parameters to validate and assess how effective the two channels are in terms of solely capturing the separate emissions. The emission in both channels was then captured under these parameters in the combination slide as a z stack. The z stacks were then deconvoluted and processed in ImageJ to single images. The Coloc2 analysis program in ImageJ was then used to assess and quantify the pixel overlap between **1**’s luminescence and the organelle-stained fluorescence as a Pearson’s correlation coefficient (0–1).

### 4.11 Dark Toxicity Assay

Cells at various densities were plated into 6 well dishes and incubated overnight. Solutions of **1** at varying concentrations were made alongside DMSO vehicle controls for the untreated conditions. The DMSO % in each solution was made equal to the DMSO concentration used in the respective **1** solution. Each well was drained of its media, and 1 mL of **1** solution (or DMSO control) was added to each dish over a range of doses. The dishes were then incubated for 2 h to allow for cellular uptake and any cytotoxic effects to occur. After the given time, **1**-containing media was drained, and each well was carefully replenished with 2 mL of fresh media. The dishes were then incubated for varying amounts of time to allow colonies to form. For dark toxicity assays, the procedure was performed to ensure that any light exposure was kept to a minimum with the Laminar Flow Cabinet light being switched off during drugging. After 10-14 days, the media was drained from each well, followed by staining with methylene blue solution (4% in 7:3 ethanol/water). After 30 min, the staining solution was removed, and the wells were carefully washed to remove excess solution before being dried. After drying, the number of colonies (>50 cells/colony) was counted in each well to form a PS dose survival curve.

### 4.12 Light Toxicity Assay

Cells were plated as 1 mL of 60,000–100,000 cells/well (cell line dependent) into wells of a 12-well dish and allowed to adhere overnight. Solutions of varying **1** concentration and DMSO vehicle control were made. The DMSO % in each solution was made to equal that of the respective **1** dose. Each well was then drained of its media, followed by addition of 1 mL of **1** (or DMSO vehicle control) to give a range of doses in one 12-well plate. The dish was then incubated for 2 h to allow for cellular uptake and dark toxicology effects to occur. After the given time, the **1**-containing medium was drained, and each well was washed with PBS (3 × 1 mL). 150 μL portion of trypsin–EDTA was added to each well, and the cells were detached through incubation. 0.85 mL of clear media (DMEM w/o l-glutamine or phenol red) was added to each well, and the cells were resuspended. A cell count was then taken of the untreated condition to calculate the volume of cell solution corresponding to 10,000 cells. This volume was then taken out of each of the other wells and complemented with clear media to make up a 1 mL volume, which was then moved into a soda glass vial or an Eppendorf per each dosed condition and placed on ice. The desired irradiation power from the laser was confirmed by using a power meter prior to cell solution exposure. The soda glass vials were then irradiated for a given time and power under a specified wavelength of excitation. After irradiation, the cells were plated into 6 well dishes for both irradiated (soda glass vials) and non-irradiated (Eppendorf tube) conditions. The plates were then incubated at 37 °C until visible colonies were formed. Following this, the media from each well was drained and the colonies were stained with the methylene blue solution (4%, in 7:3 ethanol/water, 30 min). The methylene blue solution was then removed, and each plate was carefully washed and left to dry. The colonies were then counted in each well, and a dose survival curve was plotted.

### 4.13 Caspase3/7 Assay

20,000 cells/well were plated onto sterilized coverslips in two 24-well dishes and incubated overnight. The next day, the media was drained, and the cells were drugged [**1**, 10 μM in media or DMSO control (0.2%)] and then incubated for 2 h. All wells were then washed (3 × PBS, 1 mL) and the media was replaced with PBS (1 mL/well). Selected wells were then irradiated (405 nm, 3 min, 20 mJ s^–1^ cm^2^/well), followed by incubation at 37 °C for 30 min after re-addition of media (1 mL/well). Following this, CellEvent Caspase-3/7 reagent (7.5 μM, PBS/5% FCS) was added to selected wells (100 μL) and incubated (37 °C, 30 min). All coverslips were then washed (3 × PBS, 1 mL), fixed (4% PFA in PBS, 10 min, rt), washed (2 × PBS, 1 mL), stained with DAPI (1:1000, PBS, 5 min, rt), and then washed again (3 × PBS, 1 mL). Finally, each coverslip was mounted onto glass slides (ProLong Gold Antifade, 1 drop). The detection kit contains a fluorophore that only becomes fluorescent in the presence of activated caspase 3/7 and will emit in the green (FITC) fluorescence channel of a microscope. In each condition, 100 cells were scored as either positive or negative for the signal in the green channel.

### 4.14 Spheroid Growth Monitoring

Agarose solution was prepared through addition of agarose powder to phenol-red-free DMEM (1.5%) which was then sterilized by autoclaving. This solution was stored at 4 °C until needed. The agarose solution was then microwaved until fully melted before 100 μL was added to each well of a 96-well plate. In doing this, care was taken to ensure that no bubbles were introduced to the agar. The agar was allowed to set for 1 h at room temperature before being inverted and stored at 4 °C until needed. Before plating, the agarose-coated 96 well dishes were put into an incubator for 1 h and warmed to 37 °C. C8161 cells were grown to a suitable confluency, before being washed, trypsinized, and counted. To each agarose-coated well, 9000 cells were plated in 100 μL of DMEM and incubated for 3–5 days. Spheroid growth was monitored by light microscopy until spherical morphologies with consistent diameters were formed. Every 3 days, the media was replenished by adding 100 μL of fresh DMEM and then removing 100 μL. Care was taken to not disrupt spheroids upon their manipulation. Spheroid growth was monitored using light microscopy, whereby each well was imaged under 10× magnification. The area of each imaged spheroid was then quantified by using ImageJ to assess growth/shrinkage.

### 4.15 Spheroid Toxicity Assays

Spheroids were plated and grown to ∼500 μm diameter size with visually established heterogeneity. 16 wells of the 96-well plate were sampled for each of the 4 conditions; DMSO vehicle control/not irradiated, drugged/not irradiated, DMSO vehicle control/irradiated, and drugged/irradiated. Prior to drug incubation, each well was imaged for size analysis. **1**/DMSO control was added at 2× concentration in 100 μL to each well before 100 μL was removed to give a final 1× dose which was then incubated at 37 °C for 24 h. Each well was imaged before being washed in PBS through adding/removing 100 μL (×3) before the relevant wells were irradiated in a 2 × 2 well grid (455 nm; 30 mJ s^–1^ cm^–2^, 3 min), and spheroids were then redrugged with **1** (10 μM) or DMSO vehicle control (0.2%) and incubated for 24 h. The next day, spheroids were imaged by microscopy before a second irradiation (or not) was performed (455 nm; 30 mJ s^–1^ cm^–2^, 3 min). Spheroids were then left for 24 h and imaged again. Following this, the spheroid area was quantified in ImageJ to measured spheroid growth/s.

## Supporting Information Available

Materials and methods, ^1^H NMR, ^13^C NMR, mass spectrum, crystallographic information, spectroscopic properties, time-dependent uptake of **1**, elimination of **1**, Golgi apparatus localisation and disassembly with **1**, effect of light alone on cell survival, and cell survival of 3D spheroids treated with 455 nm PDT (PDF)
SMILE strings of compound **1** (CSV)
Crystallographic data for compound **1** (CIF)

## Supplementary Material

jm4c00869_si_002.csv

jm4c00869_si_003.cif

## Notes

Compound **1** crystal structure submitted to CCDC; CCDC 2263098. Authors will release the atomic coordinates upon article publication.

## Abbreviations

ER: endoplasmic reticulum
*p*: probability value
PDT: photodynamic therapy
PI: phototoxicity index; the ratio of LD_50_(dark)/LD_50_(light)
PS: photosensitizer
SD: standard deviation
rt: room temperature
