# Supplementary material for: Photostable Iridium(III) Cyclometallated Complex is an Efficient Photosensitizer for Killing Multiple Cancer Cell Lines and 3D Models under Low Doses of Visible Light
Source: J Med Chem. 2024 Sep 4;67(18):16157–64. doi: 10.1021/acs.jmedchem.4c00869 (PMC11440503; doi:10.1021/acs.jmedchem.4c00869)
Supplement: Supplementary file 1 — jm4c00869_si_001.pdf [file jm4c00869_si_001.pdf]

Supporting Information:

A photostable Iridium(III) cyclo-metallated complex is an efficient photosensitiser for killing of multiple cancer cell lines and 3D-models under low doses of visible light

Callum Jones[1,2], Marta Martinez-Alonso[1,2], Hannah Gagg[1], Liam Kirby[2], Julia A. Weinstein[2*], Helen E. Bryant[1*]

[1] School of Medicine and Population Health, University of Sheffield, Beech Hill Road, Sheffield, S10 2RX, UK

[2] Department of Chemistry, University of Sheffield, Sheffield, S3 7HF, UK

[*] co-corresponding authors: julia.weinstein@sheffield.ac.uk or h.bryant@sheffield.ac.uk

Transition metal complexes, iridium, light-activated, singlet oxygen, phosphorescence, Golgi, mitochondria, endoplasmic reticulum, imaging, PDT, spheroids

Table of Contents

NMR Spectra and Mass spectrometry of biological tested compound *4*

Figure S1: ^{1}H NMR for *1* 4

Figure S2: ^{13}C NMR for 1 5

Figure S3: Mass Spectrum of 1 5

Crystal structure determination of 1 .... *6*

Table S1: Crystal data for *1* 6

Figure S4: structure refinement for *1* 7

Table S2: Fractional Atomic Coordinates (×104) and Equivalent Isotropic Displacement Parameters (Å2×103) for 1. 7

Table S3: Anisotropic Displacement Parameters (Å^2×10^3) for *1* 9

Table S4: Bond Lengths for *1* 10

Table S5: Bond Angles for *1* 11

Table S6: Hydrogen Bonds for *1* 13

Table S7: Hydrogen Atom Coordinates (Å×104) and Isotropic Displacement Parameters (Å2×103) for *1* 13

Table S8: Solvent masks information for *1* 14

Spectroscopic properties of 1 .... *15*

Figure S5A The initial amplitudes of the $^{1}O_2$ emission 15

Figure S5B: Emission (λ_{exc} 410 nm) and excitation (λ_{em} 550 nm) spectra of 1. 15

Figure S5C: Emission decay of 1 (DCM, λ_{em} 510 nm, λ_{exc} 410 nm) 15

Cellular uptake of 1 .... *16*

Figure S6: Accumulation of *1* in cells with time 16

Cellular Elimination of 1 .... *17*

Figure S7: Loss of *1* from cells with time 17

Golgi apparatus localisation and disassembly by 1 .... *18*

Figure S8 Co-localisation of *1* with markers of Golgi apparatus with time post-light activation 18

Cellular survival after light alone treatments *19*

Figure S9: Clonogenic survival in response to light alone treatment 19

3D spheroids treated with 455 nm PDT..*20*
Figure S10: Representative images of C8161 spheroids 20

Materials..*21*
Table S9: Materials used..21

Cell Lines..*22*
Table S10: Cancer of origin for cell lines...22

Equipment..*23*
Table S10: Equipment used for cellular studies...23

NMR Spectra and Mass spectrometry of biological tested compound

The purity of the crystalline product >95% was confirmed by ^{1}H and ^{13}C NMR and mass spectrometry

Figure S1: ^{1}H NMR for ***1***

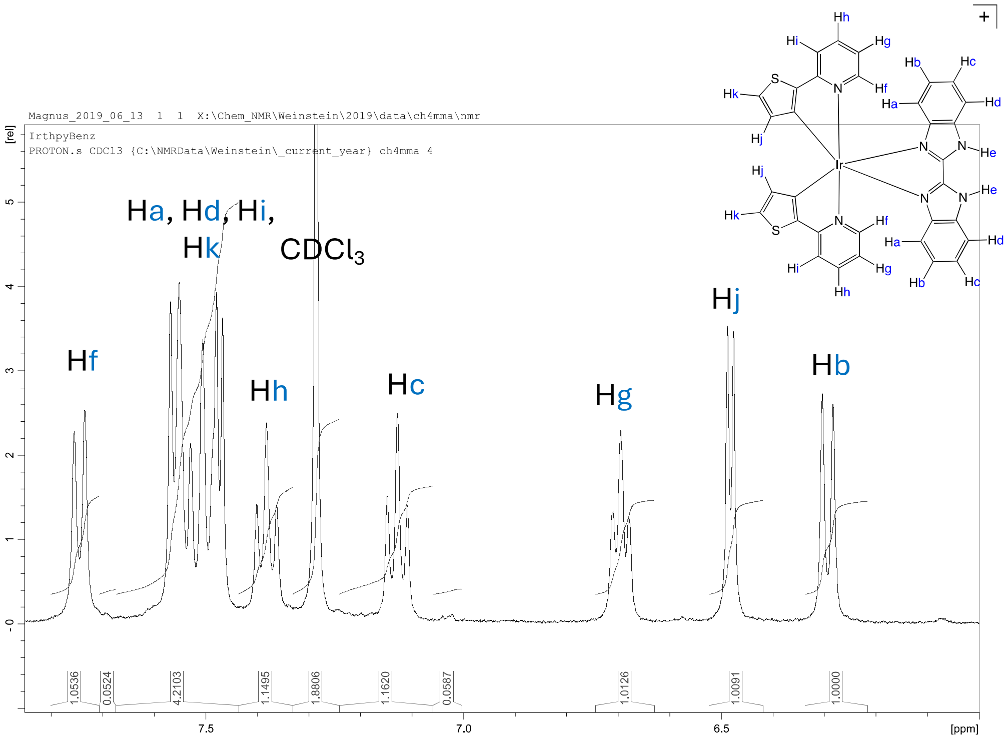

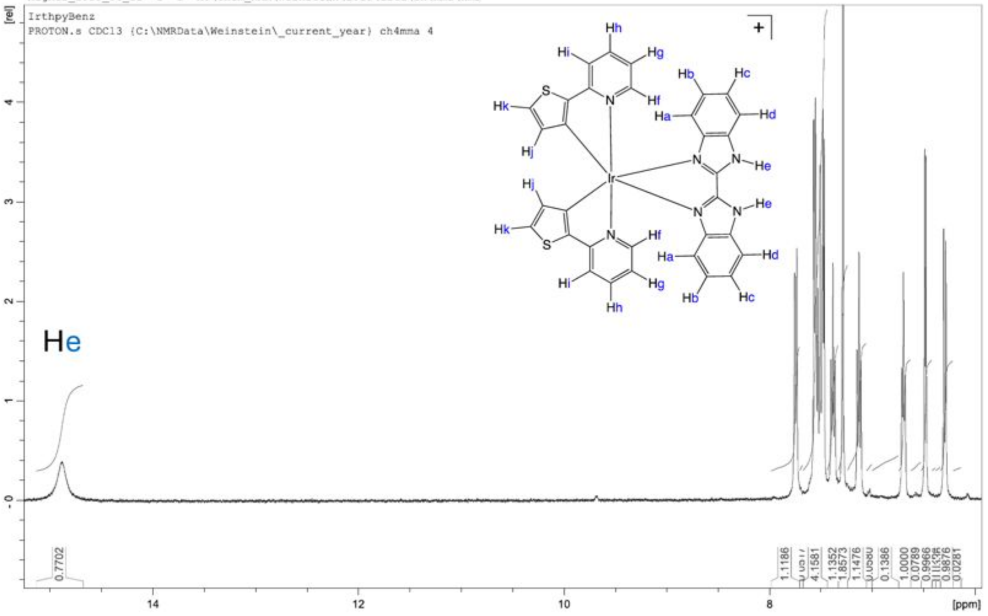

Figure S2: ^{13}C NMR for **1**

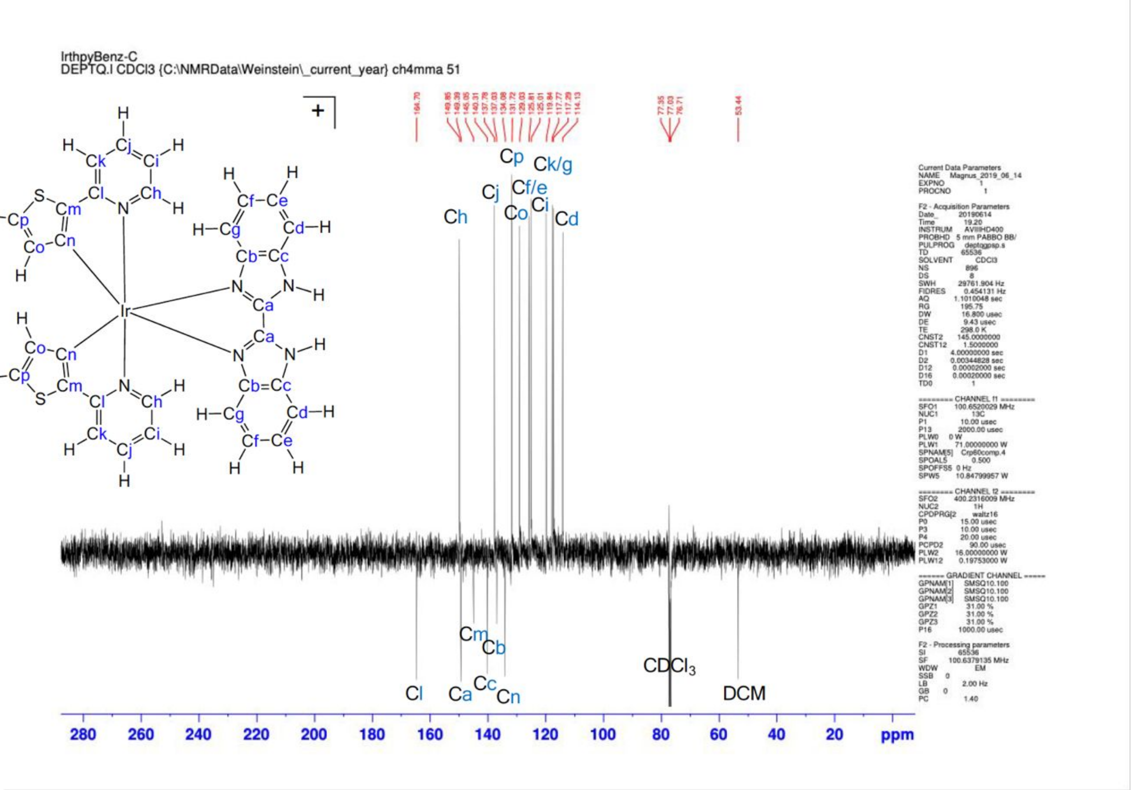

Figure S3: Mass Spectrum of 1

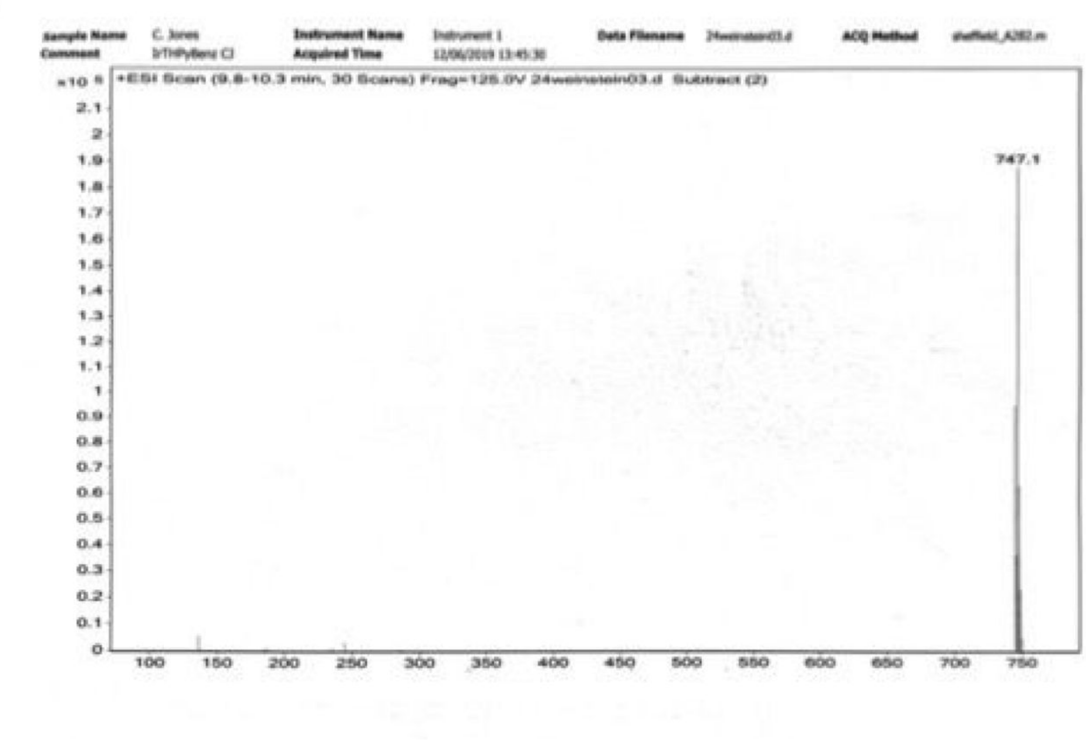

Crystal structure determination of *1*

Crystal Data for *1* (C32H22ClIrN6S2) (M =782.32 g/mol): monoclinic, space group C2/c (no. 15), a = 18.3889(4) Å, b = 18.8581(4) Å, c = 17.9797(4) Å, β = 92.2430(10)°, V = 6230.2(2) Å3, Z = 8, T = 99.99 K, μ(CuKα) = 10.595 mm-1, Dcalc = 1.668 g/cm3, 43378 reflections measured (6.716° ≤ 2Θ ≤ 133.418°), 5508 unique (Rint = 0.0462, Rsigma = 0.0231) which were used in all calculations. The final R1 was 0.0214 (I > 2σ(I)) and wR2 was 0.0443 (all data).

Table S1: Crystal data for ***1***

Table 1 Crystal data and structure refinement for 1	
Identification code	ch1jw284v_0m
Empirical formula	$C_{32}H_{22}ClIrN_6S_2$
Formula weight	782.32
Temperature/K	99.99
Crystal system	monoclinic
Space group	C2/c
a/Å	18.3889(4)
b/Å	18.8581(4)
c/Å	17.9797(4)
α/°	90
β/°	92.2430(10)
γ/°	90
Volume/Å^3	6230.2(2)
Z	8
ρ_{calc}g/cm^3	1.668
μ/mm^{-1}	10.595
F(000)	3056.0
Crystal size/mm^3	0.165 × 0.073 × 0.023
Radiation	CuKα (λ = 1.54178)
2Θ range for data collection/°	6.716 to 133.418
Index ranges	-21 ≤ h ≤ 21, -22 ≤ k ≤ 22, -21 ≤ l ≤ 21
Reflections collected	43378
Independent reflections	5508 [R_{int} = 0.0462, R_{sigma} = 0.0231]
Data/restraints/parameters	5508/1/383

Goodness-of-fit on F^2	1.069
Final R indexes [I>=2σ (I)]	R_1 = 0.0214, wR_2 = 0.0433
Final R indexes [all data]	R_1 = 0.0247, wR_2 = 0.0443
Largest diff. peak/hole / e $Å^{-3}$	0.42/-0.72

Figure S4: structure refinement for ***1***

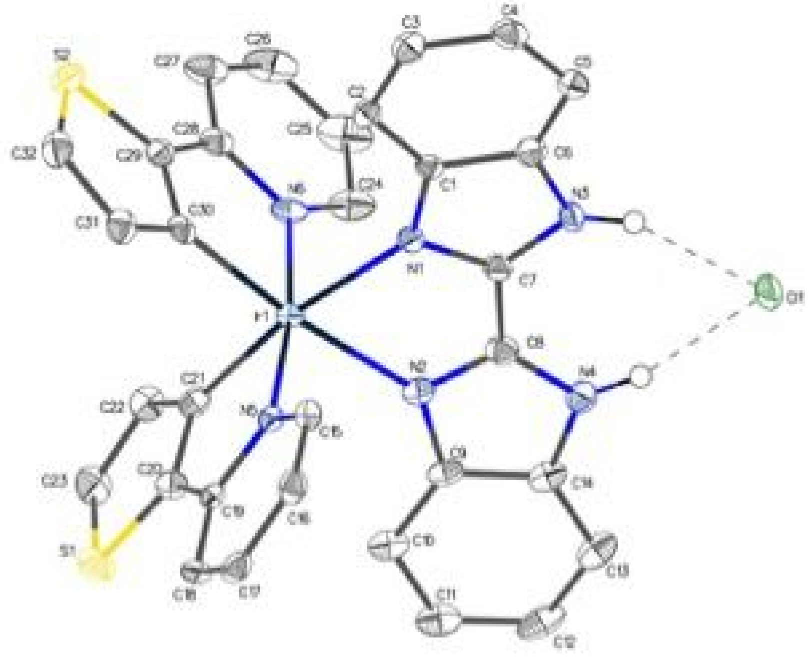

Table S2: Fractional Atomic Coordinates (×104) and Equivalent Isotropic Displacement Parameters (Å2×103) for 1.

Ueq is defined as 1/3 of of the trace of the orthogonalised UIJ tensor.

**Atom**	***x***	***y***	***z***	**U(eq)**
Ir1	7760.7(2)	5298.8(2)	4632.4(2)	12.37(4)
S1	7030.9(5)	6932.1(4)	6234.5(5)	25.97(17)
S2	9206.3(4)	6624.8(4)	3259.1(5)	24.78(17)
N1	8122.5(13)	4265.0(12)	4256.2(13)	14.4(5)
N2	6870.0(13)	4594.4(13)	4893.7(14)	16.8(5)
N3	7823.2(14)	3143.9(14)	4003.4(15)	21.1(6)
N4	6395.9(14)	3506.1(14)	4793.0(15)	19.8(5)
N5	8182.9(12)	5212.9(12)	5703.2(13)	13.9(5)

N6	7376.6(13)	5529.6(13)	3562.8(14)	17.5(5)
C1	8721.3(15)	3933.6(15)	3965.5(16)	14.1(6)
C2	9419.1(15)	4184.7(16)	3839.2(16)	15.7(6)
C3	9909.2(16)	3708.4(16)	3560.0(17)	19.5(6)
C4	9719.9(17)	3003.2(17)	3409.1(18)	22.2(7)
C5	9027.0(18)	2745.8(17)	3516.7(18)	22.3(7)
C6	8536.4(16)	3223.3(16)	3805.3(17)	18.5(6)
C7	7616.7(16)	3769.7(16)	4283.4(17)	17.3(6)
C8	6947.3(16)	3941.2(17)	4637.7(17)	19.3(6)
C9	6220.4(15)	4586.9(16)	5258.8(16)	16.9(6)
C10	5870.9(16)	5117.0(17)	5657.4(17)	20.4(7)
C11	5227.5(17)	4930.6(19)	5981.6(19)	25.4(7)
C12	4931.7(17)	4246.8(19)	5908.5(19)	28.2(8)
C13	5263.3(16)	3722.1(18)	5516.1(18)	23.6(7)
C14	5918.6(16)	3906.2(17)	5198.0(17)	19.5(6)
C15	8673.7(15)	4727.5(16)	5943.0(16)	15.8(6)
C16	8932.2(16)	4699.6(17)	6667.6(17)	19.5(6)
C17	8679.3(17)	5179.9(16)	7177.5(17)	20.7(7)
C18	8181.7(16)	5694.1(16)	6941.1(16)	18.1(6)
C19	7943.0(15)	5712.6(15)	6194.0(16)	14.8(6)
C20	7467.5(16)	6222.3(16)	5835.4(17)	18.4(6)
C21	7319.9(15)	6161.2(16)	5082.7(17)	17.4(6)
C22	6846.0(16)	6715.9(17)	4826.3(19)	21.5(7)
C23	6650.6(18)	7158.6(17)	5379(2)	26.8(7)
C24	6756.0(17)	5287(2)	3240.2(18)	27.4(7)
C25	6530(2)	5476(2)	2530(2)	35.9(9)
C26	6955(2)	5936(2)	2129.6(19)	33.8(9)
C27	7591.9(19)	6188.5(18)	2449.5(19)	27.7(8)
C28	7809.9(17)	5973.6(16)	3167.9(17)	19.0(6)
C29	8473.5(16)	6143.6(15)	3566.3(18)	18.0(6)
C30	8591.4(15)	5877.2(15)	4278.2(16)	15.6(6)
C31	9292.0(16)	6078.0(15)	4571.6(19)	19.9(6)

C32	9678.8(17)	6476.3(17)	4088(2)	24.4(7)
Cl1	8382.8(5)	2968.9(5)	5861.4(5)	36.8(2)

Table S3: Anisotropic Displacement Parameters (Å^2×10^3) for ***1***
The Anisotropic displacement factor exponent takes the form: -2π^2[h^{2}a*^{2}U$_{11}$+2hka*b*U$_{12}$+…].

**Atom**	**U$_{11}$**	**U$_{22}$**	**U$_{33}$**	**U$_{23}$**	**U$_{13}$**	**U$_{12}$**
Ir1	11.16(6)	15.28(7)	10.69(6)	-0.34(5)	0.57(4)	1.19(5)
S1	30.3(4)	23.0(4)	25.2(4)	-5.4(3)	7.1(3)	7.0(3)
S2	29.0(4)	19.6(4)	26.7(4)	2.6(3)	13.3(3)	-0.9(3)
N1	15.1(12)	15.6(12)	12.6(12)	-1.6(10)	0.1(9)	-2.3(10)
N2	14.3(12)	21.5(14)	14.7(13)	1.3(10)	2.2(9)	1.1(10)
N3	19.8(13)	18.0(13)	26.0(15)	-9.1(11)	7.9(11)	-6.7(10)
N4	15.5(12)	22.5(14)	21.7(14)	-1.1(11)	2.2(10)	-3.4(11)
N5	14.2(11)	14.4(12)	13.2(12)	-0.5(10)	2.0(9)	-3.1(10)
N6	14.5(12)	24.9(14)	13.2(13)	-0.7(10)	1.9(10)	6.9(10)
C1	16.5(14)	15.8(14)	9.9(14)	0.4(11)	0.8(11)	1.3(11)
C2	16.1(14)	16.0(14)	15.1(15)	2.8(12)	-0.1(11)	0.0(12)
C3	14.5(14)	21.6(16)	22.5(17)	1.9(13)	1.5(12)	-0.2(12)
C4	23.9(16)	23.1(16)	20.1(17)	-1.3(13)	5.3(13)	6.3(13)
C5	28.0(17)	18.6(16)	20.9(17)	-3.0(13)	8.9(13)	-0.1(13)
C6	18.7(15)	21.3(16)	15.6(16)	-0.4(12)	1.8(12)	-3.3(12)
C7	14.6(14)	21.9(15)	15.6(15)	-5.1(12)	3.8(11)	-3.6(12)
C8	15.8(14)	24.7(17)	17.5(16)	-2.0(13)	1.6(12)	-1.3(12)
C9	13.0(13)	25.9(17)	11.7(14)	5.4(12)	-0.3(11)	2.6(12)
C10	15.5(14)	28.8(18)	16.8(16)	3.6(13)	1.0(12)	5.4(12)
C11	19.1(16)	35.8(19)	21.6(17)	4.4(14)	2.9(13)	9.9(14)
C12	15.0(15)	43(2)	27.2(18)	15.2(16)	6.0(13)	4.1(14)
C13	14.2(15)	30.8(18)	25.9(18)	8.9(14)	-0.3(13)	-1.1(13)
C14	12.6(14)	27.7(17)	18.2(16)	5.0(13)	0.3(12)	2.0(12)

C15	16.6(14)	13.2(14)	17.6(15)	0.2(12)	0.2(11)	-0.6(12)
C16	20.7(15)	17.9(15)	19.5(16)	2.5(13)	-3.8(12)	-3.4(13)
C17	26.2(16)	21.9(16)	13.8(15)	4.6(12)	-2.3(12)	-9.0(13)
C18	23.3(16)	20.6(16)	10.6(15)	-2.9(12)	3.5(12)	-4.6(12)
C19	15.0(14)	14.9(14)	14.9(15)	-0.7(11)	4.9(11)	-4.8(11)
C20	17.8(15)	19.6(15)	18.1(16)	-0.9(12)	4.0(12)	0.5(12)
C21	12.3(14)	20.6(16)	19.4(16)	1.9(12)	3.1(11)	-1.4(12)
C22	17.9(15)	21.7(16)	24.9(17)	1.9(13)	0.8(13)	3.9(12)
C23	25.3(17)	19.4(16)	36(2)	0.4(14)	6.0(15)	9.0(13)
C24	14.6(15)	48(2)	19.9(17)	-2.0(16)	0.8(12)	1.9(15)
C25	24.0(17)	61(3)	22.3(19)	-4.0(17)	-6.5(14)	10.4(17)
C26	38(2)	48(2)	15.2(17)	3.9(16)	-5.0(15)	20.7(18)
C27	36.1(19)	28.6(18)	18.8(17)	2.5(14)	5.3(14)	14.5(15)
C28	22.3(15)	17.5(15)	17.5(16)	0.9(12)	5.0(12)	8.8(12)
C29	20.3(15)	12.4(14)	21.9(17)	0.0(12)	8.5(12)	5.3(12)
C30	17.9(14)	11.4(13)	17.6(15)	-2.1(12)	2.4(12)	5.3(11)
C31	19.1(15)	15.3(15)	25.5(17)	0.0(13)	1.0(13)	3.0(12)
C32	17.3(15)	18.9(16)	37(2)	-4.7(14)	6.9(14)	-1.4(12)
Cl1	41.3(5)	29.5(4)	41.2(5)	-19.1(4)	20.5(4)	-21.5(4)

Table S4: Bond Lengths for ***1***

**Atom**	**Atom**	**Length/Å**		**Atom**	**Atom**	**Length/Å**
Ir1	N1	2.176(2)		C4	C5	1.384(5)
Ir1	N2	2.174(2)		C5	C6	1.389(4)
Ir1	N5	2.054(2)		C7	C8	1.445(4)
Ir1	N6	2.069(3)		C9	C10	1.401(4)
Ir1	C21	2.002(3)		C9	C14	1.401(4)
Ir1	C30	2.001(3)		C10	C11	1.385(4)
S1	C20	1.731(3)		C11	C12	1.404(5)

S1	C23	1.719(4)		C12	C13	1.372(5)
S2	C29	1.733(3)		C13	C14	1.398(4)
S2	C32	1.719(4)		C15	C16	1.370(4)
N1	C1	1.386(4)		C16	C17	1.382(4)
N1	C7	1.321(4)		C17	C18	1.388(4)
N2	C8	1.324(4)		C18	C19	1.397(4)
N2	C9	1.385(4)		C19	C20	1.435(4)
N3	C6	1.381(4)		C20	C21	1.375(4)
N3	C7	1.343(4)		C21	C22	1.426(4)
N4	C8	1.342(4)		C22	C23	1.357(5)
N4	C14	1.386(4)		C24	C25	1.375(5)
N5	C15	1.345(4)		C25	C26	1.389(6)
N5	C19	1.376(4)		C26	C27	1.369(5)
N6	C24	1.340(4)		C27	C28	1.398(5)
N6	C28	1.373(4)		C28	C29	1.427(4)
C1	C2	1.395(4)		C29	C30	1.384(4)
C1	C6	1.409(4)		C30	C31	1.424(4)
C2	C3	1.381(4)		C31	C32	1.369(4)
C3	C4	1.399(4)				

Table S5: Bond Angles for ***1***

**Atom**	**Atom**	**Atom**	**Angle/°**		**Atom**	**Atom**	**Atom**	**Angle/°**
N2	Ir1	N1	76.23(9)		N2	C8	N4	113.8(3)
N5	Ir1	N1	96.42(9)		N2	C8	C7	117.8(3)
N5	Ir1	N2	90.55(9)		N4	C8	C7	128.2(3)
N5	Ir1	N6	172.16(10)		N2	C9	C10	131.0(3)
N6	Ir1	N1	89.82(9)		N2	C9	C14	108.6(3)
N6	Ir1	N2	95.55(10)		C10	C9	C14	120.4(3)
C21	Ir1	N1	170.70(10)		C11	C10	C9	116.8(3)
C21	Ir1	N2	95.08(10)		C10	C11	C12	121.9(3)
C21	Ir1	N5	80.17(11)		C13	C12	C11	122.2(3)
C21	Ir1	N6	94.36(11)		C12	C13	C14	116.0(3)
C30	Ir1	N1	98.21(10)		N4	C14	C9	106.4(3)

C30	Ir1	N2	172.99(10)		N4	C14	C13	130.8(3)
C30	Ir1	N5	94.36(11)		C13	C14	C9	122.7(3)
C30	Ir1	N6	80.02(11)		N5	C15	C16	122.3(3)
C30	Ir1	C21	90.70(11)		C15	C16	C17	119.5(3)
C23	S1	C20	90.01(15)		C16	C17	C18	119.3(3)
C32	S2	C29	90.64(15)		C17	C18	C19	119.4(3)
C1	N1	Ir1	141.09(19)		N5	C19	C18	120.2(3)
C7	N1	Ir1	113.44(19)		N5	C19	C20	112.1(3)
C7	N1	C1	105.4(2)		C18	C19	C20	127.7(3)
C8	N2	Ir1	113.64(19)		C19	C20	S1	128.0(2)
C8	N2	C9	105.2(2)		C21	C20	S1	113.3(2)
C9	N2	Ir1	141.2(2)		C21	C20	C19	118.7(3)
C7	N3	C6	106.7(2)		C20	C21	Ir1	113.5(2)
C8	N4	C14	106.0(3)		C20	C21	C22	110.6(3)
C15	N5	Ir1	125.4(2)		C22	C21	Ir1	135.8(2)
C15	N5	C19	119.3(3)		C23	C22	C21	113.0(3)
C19	N5	Ir1	115.36(19)		C22	C23	S1	113.1(2)
C24	N6	Ir1	125.8(2)		N6	C24	C25	122.4(3)
C24	N6	C28	119.1(3)		C24	C25	C26	119.2(3)
C28	N6	Ir1	115.2(2)		C27	C26	C25	119.3(3)
N1	C1	C2	131.1(3)		C26	C27	C28	119.8(3)
N1	C1	C6	108.5(2)		N6	C28	C27	120.2(3)
C2	C1	C6	120.4(3)		N6	C28	C29	112.3(3)
C3	C2	C1	117.1(3)		C27	C28	C29	127.4(3)
C2	C3	C4	121.9(3)		C28	C29	S2	128.2(2)
C5	C4	C3	122.0(3)		C30	C29	S2	112.9(2)
C4	C5	C6	116.1(3)		C30	C29	C28	118.9(3)
N3	C6	C1	105.9(3)		C29	C30	Ir1	113.5(2)
N3	C6	C5	131.6(3)		C29	C30	C31	110.7(3)
C5	C6	C1	122.5(3)		C31	C30	Ir1	135.9(2)
N1	C7	N3	113.4(3)		C32	C31	C30	113.3(3)
N1	C7	C8	118.1(3)		C31	C32	S2	112.5(2)

N3	C7	C8	128.3(3)					

Table S6: Hydrogen Bonds for ***1***

**D**	**H**	**A**	**d(D-H)/Å**	**d(H-A)/Å**	**d(D-A)/Å**	**D-H-A/°**
N3	H3	Cl1[1]	0.88	2.24	3.070(3)	157.3
N4	H4	Cl1[1]	0.879(18)	2.20(2)	3.054(3)	165(3)

Table S7: Hydrogen Atom Coordinates (Å×104) and Isotropic Displacement Parameters (Å2×103) for ***1***

**Atom**	***x***	***y***	***z***	**U(eq)**
H3	7555.85	2758.4	3955.56	25
H4	6400(20)	3058(11)	4660(20)	31(10)
H2	9551.51	4662.88	3940.9	19
H3A	10388.64	3864.19	3467.68	23
H4A	10077.69	2691.38	3227.33	27
H5	8894.64	2270.59	3400.09	27
H10	6065.95	5582.17	5703.07	24
H11	4979.18	5275.27	6261.59	31
H12	4487.29	4143.93	6138.2	34
H13	5059.58	3261.01	5463.78	28
H15	8847.23	4392.02	5598.01	19
H16	9282.69	4352.94	6818.31	23
H17	8844.09	5158.34	7684.42	25
H18	8005.31	6030.08	7283.95	22
H22	6684.17	6769.81	4321.15	26
H23	6335.81	7553.34	5300.52	32
H24	6461.51	4974.01	3512.49	33
H25	6088.53	5292.8	2314.85	43
H26	6806.14	6075.4	1639.16	41
H27	7884.58	6508.58	2183.81	33
H31	9472.86	5948.39	5054.75	24
H32	10155.31	6651.15	4198.88	29

Table S8: Solvent masks information for ***1***

Number	X	Y	Z	Volume	Electron count	Content
1	0.000	0.156	0.750	150.7	51.2	CH_2Cl_2
2	0.000	0.214	0.250	68.2	6.1	?
3	0.000	0.844	0.250	150.7	50.5	CH_2Cl_2
4	0.000	0.786	0.750	68.2	6.1	?
5	0.500	0.344	0.250	150.7	50.5	CH_2Cl_2
6	0.500	0.656	0.750	150.7	51.2	CH_2Cl_2
7	0.500	0.286	0.750	68.2	6.1	?
8	0.500	0.714	0.250	68.2	6.1	?

Spectroscopic properties of 1

Figure S5A The initial amplitudes of the $^1O_2$ emission
Taken at 1270 nm as a function of excitation laser power (355 nm, 8 ns) of perinapthenone and **1** in air-saturated CH_3CN.

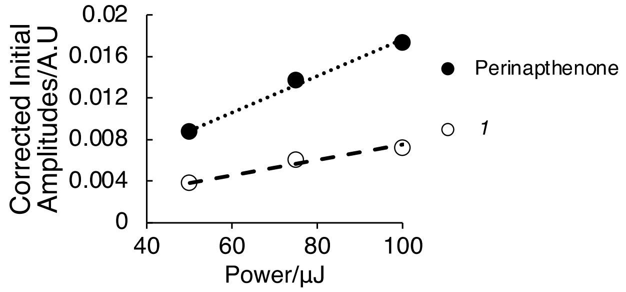

Figure S5B: Emission (λ_{exc} 410 nm) and excitation (λ_{em} 550 nm) spectra of **1**.
Taken in DCM under air and argon.

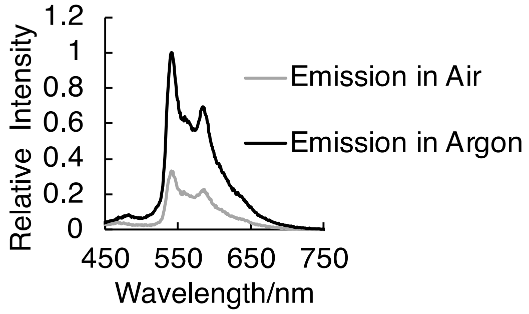

Figure S5C: Emission decay of **1** (DCM, λ_{em} 510 nm, λ_{exc} 410 nm)
Taken under air and argon. The first, <1 ns decay, is due to excitation laser scattering.

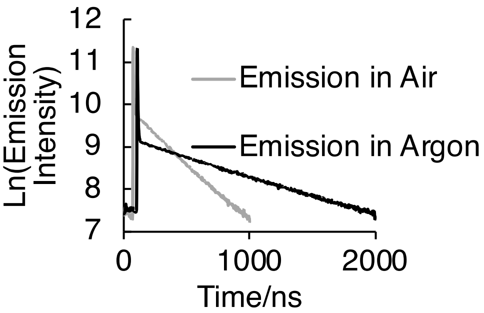

Cellular uptake of *1*

Figure S6: Accumulation of ***1*** in cells with time

Representative images of fluorescence microscopy of EJ cells following treatment of 1 (30 µM) after increasing incubation times (λ_{exc} 350 – 400 nm, λ_{em} 570 nm). Cells were incubated with **1**, fixed and then imaged on a fluorescence widefield microscope. Scale bar = 20 µm

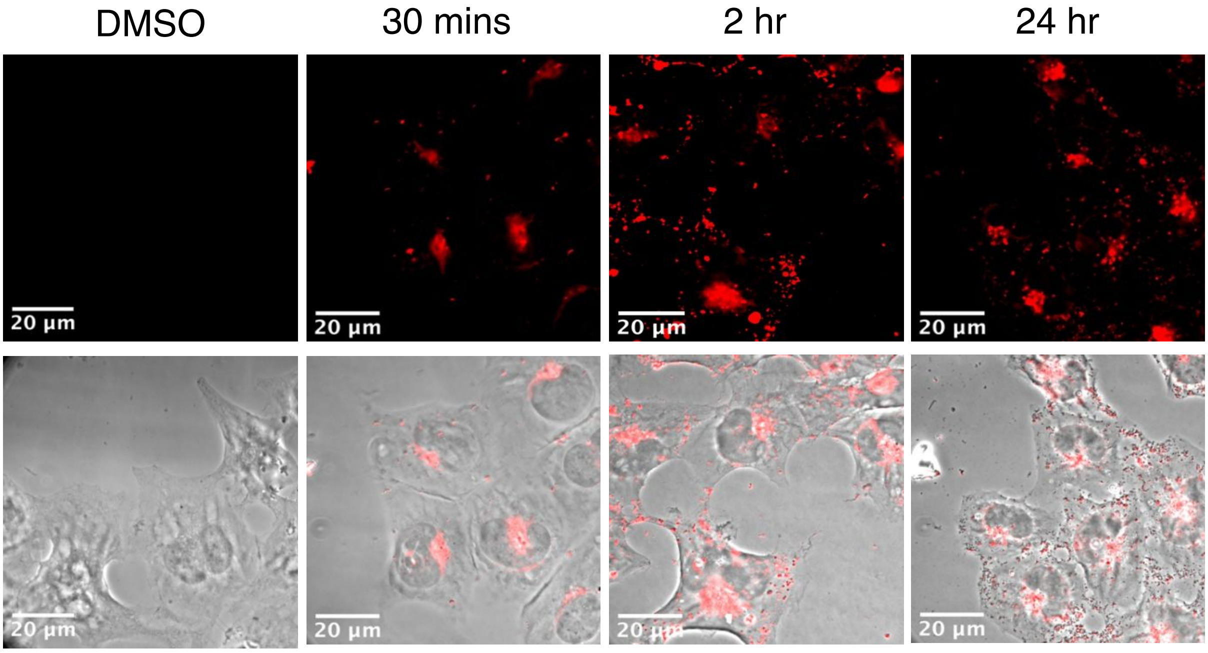

Cellular Elimination of *1*

Figure S7: Loss of *1* from cells with time

Representative images of **1** incubated with EJ cells (20 µM, 2 hrs), washed with PBS and then incubated with fresh undrugged media. Immediately after washing, cells were fixed and imaged (λ_{exc} 350 – 400 nm) and then again at 24 hr and 48 hr. Scale bar = 20 µm

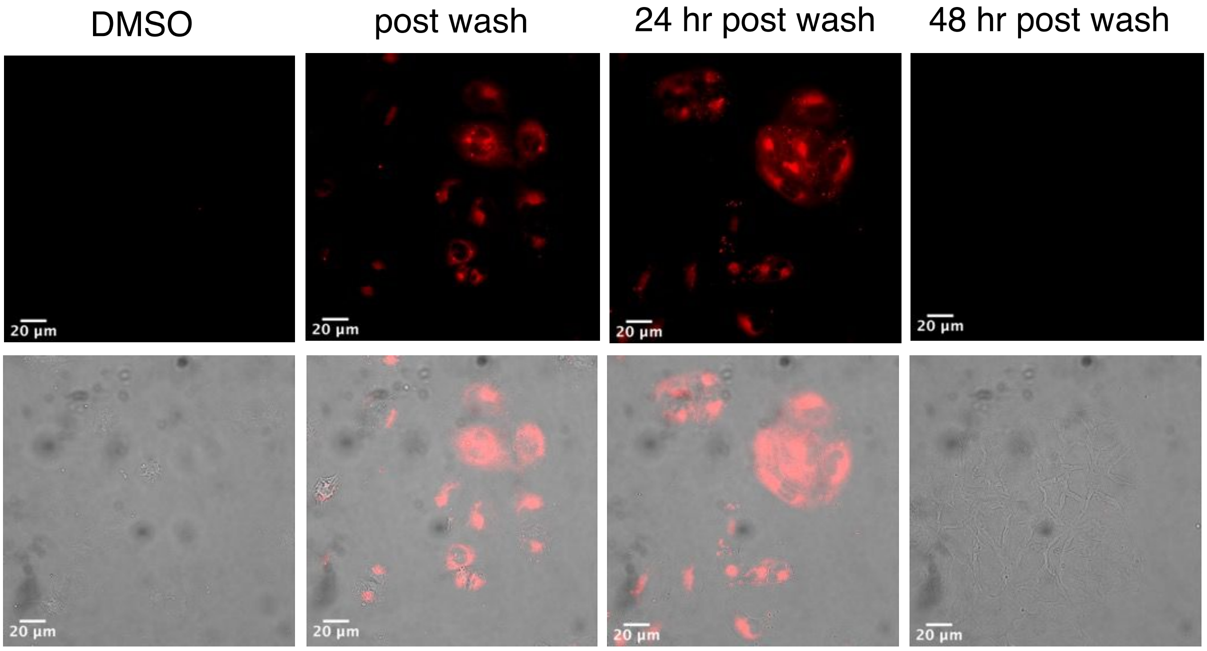

Golgi apparatus localisation and disassembly by *1*

1 co-localised with the Golgi apparatus and by 15-30 mins post-light exposure the distribution of the marker was disrupted into a pattern characteristic of Golgi disassembly.

Figure S8 Co-localisation of *1* with markers of Golgi apparatus with time post-light activation

Representative images of EJ cells following 2 hour incubation with 30 µM **1** (red), co-localised with Golgi specific 58K-9 protein (green) at increasing times post irradiation, scale bar = 20 µm. Co-localization expressed as a PCC (Pearson's Correlation Coefficient) N > 15 cells. Zoomed sections are shown as insets.

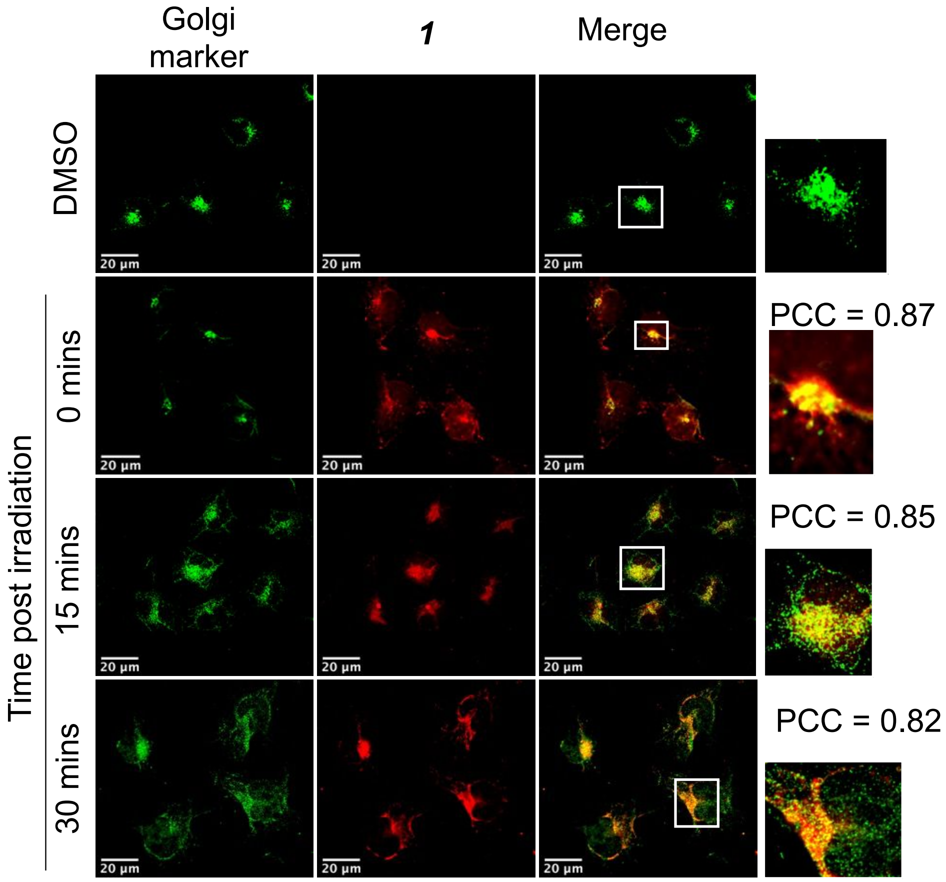

Cellular survival after light alone treatments

No difference in survival was seen following treatment with light alone.

Figure S9: Clonogenic survival in response to light alone treatment
EJ cells were treated with 405 nm (20 $mJs^{-1}cm^{-2}$, 3 mins) or 455 nm (30 $mJs^{-1}cm^{-2}$, 3 mins) irradiation, then allowed to form colonies which were counted and plotted relative to non-treated (dark) control cells. In each case the mean and SDs of ≥ 3 independent repeats are shown. ns = none significant Student's T test (two-tailed, paired)

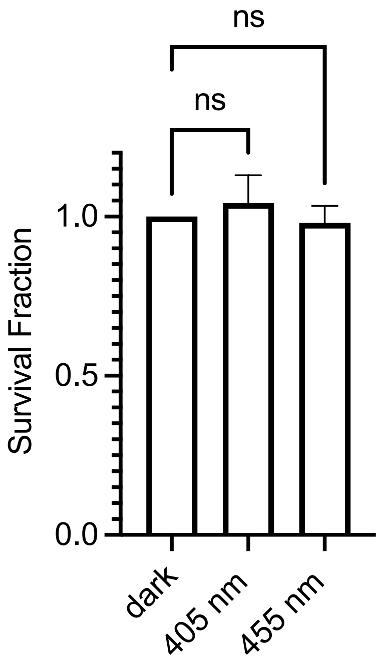

3D spheroids treated with 455 nm PDT

Figure S10: Representative images of C8161 spheroids
Images were taken following single (48 h) or double (72 h) 455 nm light treatment (30 mJs-1cm-2, 3 mins) in presence or absence of 1. Scale bar = 500 µM

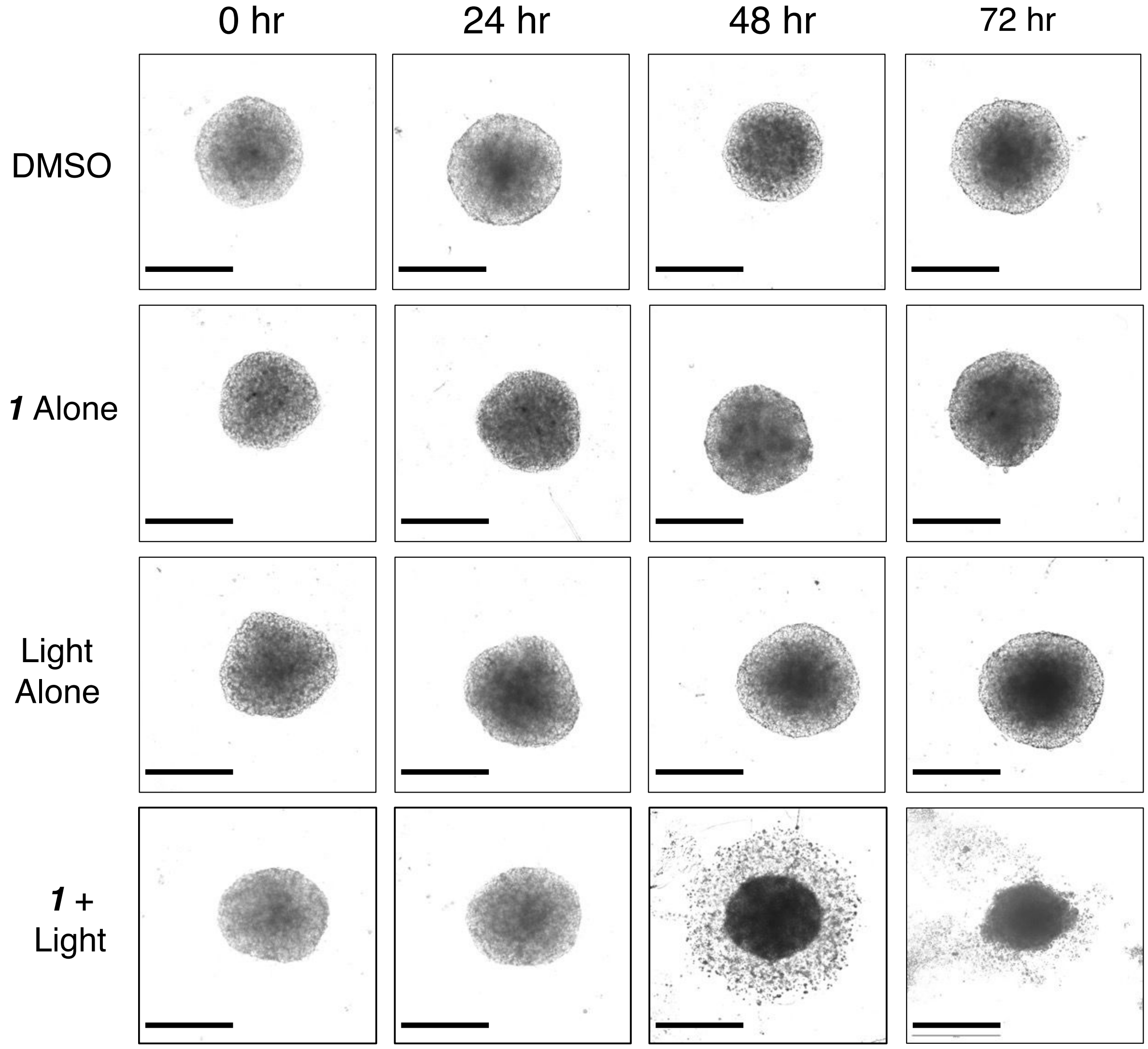

Materials

Table S9: Materials used

Material	Full name	Vendor
γH2AX (Ser139) monoclonal antibody		Millipore
58K-9 Golgi protein antibody		Abcam
Agarose powder	Agarose (Protein Electrophoresis Grade)	Fisher Scientific
Anti-mouse 488		Invitrogen
Anti-rabbit 488		Invitrogen
BSA	Bovine serum albumin	Sigma-Aldrich
Calnexin monoclonal antibody		ThermoFisherScientific
CellEvent™ Caspase 3/7 Green Detection Reagent		ThermoFisher Scientific
Chloroquine	Chloroquine diphosphate salt	Sigma Aldrich
Clear DMEM	Dulbecco's Modified Eagle's Medium. DMEM 4.5 g/L glucose without L-glutamine and phenol red	Lonza
Cytochrome C monoclonal antibody		ThermoFisher Scientific
DCM	Dichloromethane	Sigma Aldrich
DMEM	Dulbecco's Modified Eagle's Medium. DMEM 4.5 g/L glucose, glutamine, phenol red	Sigma-Aldrich
DMSO	Dimethyl sulfoxide	Sigma Aldrich
DMSO	Dimethyl sulfoxide	Lonza
EDTA	Ethylenediaminetetraacetic acid	Sigma Aldrich
EtOH	Ethanol	Sigma Aldrich
FCS	Foetal calf serum	Biosera

Hexane		Sigma Aldrich
IMMU-mount	Thermo Scientific™ Shandon™ IMMU-mount™	Life Technologies Ltd, Paisley, UK
Methylene blue		ThermoFisher Scientific
PBS	Phosphate buffered saline solution	Oxoid
PFA	Paraformaldehyde solution	ThermoFisher Scientific
Precept		Johnson and Johnson medical limited
Pro-long gold mountant	Molecular Probes Pro Long Gold Antifade Mountant	Fisher Scientific
Protein Assay Dye Reagent Concentrate		BIO-RAD
Tetraethyl ammonium chloride		Alfa Aesar
Triton X-100		ThermoFisher Scientific
Trypsin		Sigma-Aldrich
Valinomycin	Valinomycin ≥ 98% (TLC), ≥ 90% HPLC	Sigma-Aldrich

Cell Lines

Cells were routinely cultured and grown in suitable medium (DMEM, 10 % FCS) unless otherwise stated and incubated at 37 °C, 5% CO_2. Cells were grown in a T-75 flask until reaching a suitable confluency 80 – 100 %, depending on the cell type.

Table S10: Cancer of origin for cell lines

Cell lines	Cancer of origin
A375	Human epithelial malignant melanoma
C1861	Human cutaneous melanoma
EJ	Human epithelial bladder carcinoma
OPSCC72	Human oropharynx small cell carcinoma

Equipment

Table S10: Equipment used for cellular studies

Name	Company
Cell culture centrifuge – Heraeus MegaFuge 16	ThermoFisher Scientific
Colony counter	Stuart scientific
Haemocytometer	Hawksley
Incubator	Weis Gallenkamp
Nikon dual-cam widefield live-cell system	Nikon
Waterbath	Grant Instruments
Plate reader	ThermoScientific Multiskan FC
